# Intraoperative Hearing Monitoring Using ABR and TT-ECochG and Hearing Preservation during Vestibular Schwannoma Resection

**DOI:** 10.3390/jcm13144230

**Published:** 2024-07-19

**Authors:** Kazimierz Niemczyk, Izabela Pobożny, Robert Bartoszewicz, Krzysztof Morawski

**Affiliations:** 1 Department of Otorhinolaryngology Head and Neck Surgery, Medical University of Warsaw, 02-091 Warsaw, Poland; 2 Department of Otorhinolaryngology, Institute of Medical Sciences, University of Opole, 45-040 Opole, Poland

**Keywords:** auditory brainstem responses, acoustic neuroma, action potential, cochlear nerve, electrocochleography, hearing loss, hearing preservation, intraoperative monitoring, vestibular schwannoma

## Abstract

**Background:** Quick and appropriate diagnostics and the use of intraoperative monitoring (IM) of hearing during vestibular schwannoma (VS) resection increase the likelihood of hearing preservation. During surgery, various methods of IM can be used, i.e., auditory brainstem responses (ABRs), transtympanic electrocochleography (TT-ECochG), and direct cochlear nerve action potentials. The aim of the study was to evaluate the prognostic values of IM of hearing using ABR and TT-ECochG in predicting postoperative hearing preservation and to evaluate relationships between them during various stages of surgery. **Methods:** This retrospective study presents the pre- and postoperative audiological test results and IM of hearing records (TT-ECochG and ABR) in 75 (43 women, 32 men, aged 18–69) patients with diagnosed VS. **Results:** The preoperative pure tone average hearing threshold was 25.02 dB HL, while after VS resection, it worsened on average by 30.03 dB HL. According to the American Academy of Otolaryngology–Head and Neck Surgery (AAO—HNS) Hearing Classification, before and after (pre/post) surgery, there were 47/24 patients in hearing class A, 9/8 in B, 2/1 in C, and 17/42 in D. In speech audiometry, the average preoperative speech discrimination score at an intensity of 60 dB SPL was 70.93%, and after VS resection, it worsened to 38.93%. The analysis of electrophysiological tests showed that before the tumor removal the I–V ABR interlatencies was 5.06 ms, and after VS resection, it was 6.43 ms. **Conclusions:** The study revealed correlations between worse postoperative hearing and changes in intraoperatively measured ABR and TT-ECochG. IM of hearing is very useful in predicting postoperative hearing in VS patients and increases the chance of postoperative hearing preservation in these patients.

## 1. Introduction

Vestibular schwannomata (VS) are histologically benign, slow-growing tumors developing from the Schwann sheath. Most often, they occur unilaterally, but sometimes they may appear on both sides and are usually associated with the clinical picture of type 1 or type 2 neurofibromatosis [1]. The treatment strategy in the case of VS depends on various factors (e.g., tumor size, patient’s age, condition of the hearing organ) influencing the final choice of therapy among three possible modes: microsurgery, stereotactic radiotherapy, or observation [2,3,4,5]. According to the recommendations of the National Institutes of Health Consensus Development Conference on Acoustic Neuroma in 1991 [3], the best VS treatment method is microsurgery. Its goal is to completely remove the tumor while preserving the neurological functions of adjacent structures. VS microsurgery by the middle fossa approach (MFA) or posterior fossa approach (PFA) enables complete VS resection and hearing function preservation. To maximize the likelihood of hearing preservation with both of these surgical approaches, intraoperative hearing monitoring (IM) is used [6]. The most commonly used electrophysiological tests for IM of hearing are auditory brainstem responses (ABR) [7,8,9,10,11,12,13,14,15,16,17,18,19,20,21,22,23,24,25,26], transtympanic electrocochleography (TT-ECochG) [7,8,10,11,12,16,17,27,28], and direct cochlear nerve action potential (CNAP) [7,9,10,12,13,15,16,17,19,20,22,23,24,25,26,27,29,30,31]. Each of the mentioned electrophysiological techniques has advantages and disadvantages. The most frequently used test is ABR, which is a non-invasive far-field technique, but recording auditory potentials requires several hundred averages to make the recording repeatable, legible, and interpretable. Consequently, the obtained result usually has a time delay of several dozen seconds at critical stages of the surgery, so it is difficult to say that the test is carried out in real time, which may turn out to be a critical element for preserving hearing [7,8,9,11]. TT-ECochG and CNAP are invasive and technically more difficult to perform, which is why some surgeons do not use them for IM of hearing. With readings from the near field, the amplitudes of the auditory potentials are higher, and usually, averaging several dozen responses is sufficient to obtain clear, repeatable recordings. It can therefore be said that IM of hearing takes place in the real-time domain [7,8,9,10,11,12,13,15,16,17,18,19,20,22,23,24,25,26,27,28,29,30,31].

The final decision regarding preference of audiological technique for IM of hearing during VS resection depends on the tradition of the department, the individual experience of the audiologist, and the audiological equipment used in the surgical room. Another important factor that guarantees final success is effective co-operation between the surgeon removing tumor and the audiologist intraoperatively monitoring hearing. Rapid information about the status of hearing during VS removal, as provided by the audiologist, and the correct reaction of the surgeon is an element of this co-operation that increases the chances of postoperative hearing preservation. In the majority of otolaryngology centers where vestibular schwannomata are operated upon, hearing is monitored intraoperatively by one of the aforementioned electrophysiological techniques. The authors of this paper monitored hearing during VS resection using two techniques simultaneously: ABR and TT-ECochG. Such a strategy for IM of hearing makes it possible to present different mutual dependences between various intraoperative parameters characterizing ABR and TT-ECochG and their correlation with VS size and postoperative hearing status. The authors of this paper have a great experience in intraoperative monitoring of hearing during various types of surgeries, including vestibular schwannoma removal [11,27,28] and ossiculoplasty [32,33]. Additionally, they have experience in animal models of intraoperative hearing monitoring during induced ischemia of the cochlea using TT-ECochG, distortion product otoacoustic emissions (DPOAE), and the laser Doppler technique [34,35].

The authors of this study evaluated the prognostic values of IM of hearing using ABR and TT-ECochG in predicting postoperative hearing preservation and analyzed the relationships between them during IM at various stages of surgery.

## 2. Material and Methods

### 2.1. Patients’ Presentation

In our University Hospital, VS surgery is performed at Department of Otolaryngology or Department of Neurosurgery. All small VS with preserved hearing included in this study were resected through the MFA by an otolaryngologist, always by the first author of this paper, while bigger (usually > 25 mm) tumors with preserved hearing were removed in the Neurosurgery Department by the neurosurgeons’ team through the posterior fossa approach. Thus, in this paper, we focus only on cases of VS resected via the MFA. All included patients were intraoperatively monitored by the same audiologists that always used two electrophysiological techniques simultaneously: ABR and TT-ECochG. A total of 75 patients were included in the study (43 women (57.33%) and 32 men (42.66%)). The age of the patients ranged from 18 to 69 years (average 46.44; median 48.00, first quartile (Q1) 36.00; third quartile (Q3) 58.00; standard deviation (SD) 14.04). All VS patients were diagnosed and confirmed clinically and radiologically via magnetic resonance imaging (MRI) with contrast. The tumors filled the internal auditory canal (IAC), not exceeding 2.5 cm in the longitudinal axis of the IAC, and in some cases protruded beyond the border of the IAC by no more than 1.5 cm. 

### 2.2. Audiological Test Methodology

All presented patients had audiological tests performed 1–7 days before surgery and up to 30 days after VS resection. In the case of deafness to individual frequencies in pure tone audiometry (TA), a value of 130 dB HL (decibel hearing loss) was assumed as the maximum for statistical calculation purposes. The obtained results were calculated, analyzed, and classified according American Academy of Otolaryngology—Head and Neck Surgery (AAO—HNS) Hearing Classification [36]. Preoperative (preop) and postoperative (postop) hearing were evaluated using TA tested in the 125–8000 Hz frequency band, and speech audiometry (SA) was used to evaluate speech discrimination in monosyllabic verbal tests. For statistical calculations, the preop and postop hearing threshold was expressed according to the following formula: (0.5 kHz + 1.0 kHz + 2.0 kHz + 3.0 kHz)/4)(PTA-4), while SA was presented as a percentage of correctly repeated monosyllabic words presented at an intensity of 60 dB SPL (sound pressure level); (SA—60 dB SPL). Postop hearing changes in TA were presented as the difference between postop and preop PTA-4 (post–pre PTA-4). Postop hearing changes in SA were presented as the difference between postop and preop SA-60 dB SPL (post–pre SA—60 dB SPL). Worsened postop hearing was evaluated according to AAO—HNS Hearing Classification and was also represented as the difference between postop and preop AAO—HNS. PTA-4 in 71 patients did not exceed 50 dB HL, and in SA—60 dB SPL, speech discrimination was at least 50%. Four patients, despite a slightly worse hearing threshold and speech discrimination, were also qualified for VS resection through the MFA due to a tumor of grade I or II on the Koos Grading Scale [37]. Therefore, according to the American Academy of Otolaryngology—Head and Neck Surgery (AAO—HNS) Hearing Classification [36], 47 patients were classified as class A, 9 as class B, 2 as hearing class C, and 17 as hearing class D. All patients were qualified for tumor resection via the MFA with accompanying IM of hearing. 

### 2.3. Methodology of Intraoperative Hearing Monitoring

To perform hearing IM during VS removal, ABR and TT-ECochG tests were used.

A two-channel Smart Box device with Smart EP software v. 2.70 (Intelligent Hearing Systems, Miami, FL, USA) was used to perform IM of hearing. In each case, at the beginning of the surgery, the needle electrode (−) for TT-ECochG and ABR responses was inserted through the posteroinferior quadrant of the tympanic membrane and supported by the promontory. The grounding needle electrode is normally placed in the midline at the border of the forehead and scalp, and the reference (+) needle electrode is placed on the top of the head. The acoustic stimulus was an 80 dB nHL click with alternating polarization delivered through ER3 insert earphones (Etymotic Research, Elk Grove Village, IL, USA). Each time the stimulus presentation rate was 21.17/s, the number of averaged samples ranged between 64 and 256, thus enabling clear and repeatable responses to be obtained with both TT-ECochG and ABR. The parameters of acquisition were typical: amplification: 100 k gain; filtering: 30–3000 Hz; and time window of the analyses: 12.8 ms.

In TT-ECochG, the latency and amplitude of the action potential (AP; AP—Lat, AP—Amp) were assessed. In the case of lack of AP due to the loss of auditory functions after AS resection, the AP—Lat value for statistical calculations was assumed to be 5.6 ms, i.e., the last possible measurable AP peak, and for the AP-Amp, the value was 0.00 µV. In ABR, the latencies of waves I, III, V and the values of individual time interlatencies I–III, III–V, and I–V were assessed. In the absence of a recorded ABR response for wave V in a patient after tumor removal, a latency value of 12.8 ms was assumed for statistical calculations, as the last measurable value of the analyzed time window. Additionally, for this reason, the value of the I–V interlatencies after surgery was assumed to be 12.8 ms.

Intraoperative electrophysiological recordings were performed for the entire surgery, but for statistical purposes, recordings from 3 stages of surgery were analyzed: during the tumor approach preparation (Stage 1); during manipulation on VS and its resection (Stage 2), and after VS resection (Stage 3).

Electrophysiological responses were recorded in real time every 5–6 s with TT-ECochG and for a slightly usually twice longer time with ABR (twice the usual length of time). By combining these two techniques, it was possible to verify the condition of the hearing organ relatively quickly and inform the surgeon about any changes in the morphology of the TT-ECochG and ABR. After assembling the research group, all electrophysiological responses were subjected to detailed analyses and calculations.

### 2.4. Statistical Analyses

Due to the lack of normal data distributions, small subgroup sizes, and failure to meet other criteria for the use of parametric tests, we decided to use their non-parametric equivalents. The Wilcoxon test was used to compare changes in values observed in audiological and electrophysiological tests before and after surgery. Spearman’s rank correlation test (R) was used to assess the correlations between various parameters. The Pearson χ2 test of maximum likelihood was used to analyze the distribution of qualitative characteristics. A *p*-value < 0.05 was considered statistically significant.

## 3. Results

### 3.1. Vestibular Schwannoma Dimensions in MRI

In the presented patients, the average tumor size in the longitudinal axis was 10.26 mm (median 10.00; min–max 4.0–20.00; Q1 7.00; Q3 14.00; SD 4.77), in the horizontal axis was 6.68 mm (median 6.00; min–max 3.0–18.00; Q1 5.00; Q3 8.00; SD 2.92), and in the vertical axis was 6.52 mm (median 6.00; min–max 3.0–16.00; Q1 4.00; Q3 8.00; SD 2.90). According to the Koos Grading Scale [37], 51 patients were classified as grade I, 22 of them as grade II, and only 2 as grade III. According to the Matthies’ guidelines [38], T1 intracanal tumor occurred in 51 patients, T2 in 22, and T3 in 2 patients. In 42 patients, the VS was located on the right side, and in 33, it was located on the left.

### 3.2. Analysis of Audiological Tests before and after Vestibular Schwannoma Removal

Pre- and postoperative TA and SA were performed in all 75 patients included in the project. Table 1 presents the individual results of the statistical analyses of these audiological tests.

Pre PTA-4 values were found to range from 2.50 to 63.75 dB HL (median 22.5). After VS removal, hearing deteriorated and ranged from 10.00 to 130.00 dB HL (median 30.03). The median value of postoperative hearing was significantly worse, reaching 15 dB (Wilcoxon test: Z = 7.20; *p* < 0.001). Detailed results are presented in Table 1.

Analysis of the distribution of hearing deterioration values showed that in 31 (41.33%) patients, the postop hearing threshold for PTA-4 did not deteriorate by more than 10 dB HL, and in 55 (73.33%) patients, it did not deteriorate by more than 30 dB HL. Among the remaining 20 patients whose hearing threshold deteriorated by more than 30 dB HL, there were also 13 (17.33%) patients who completely lost hearing functions after VS resection.

**Figure 1:**
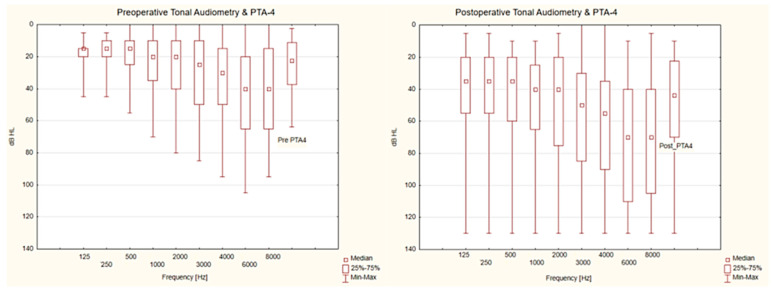
Preoperative and postoperative tonal audiometry as well as preoperative and postoperative tonal audiometry calculated using the following formula: (0.5 kHz + 1.0 kHz + 2.0 kHz + 3.0 kHz)/4)(PTA-4). dB HL: decibel hearing level; Hz: Hertz; PTA-4: tonal audiometry calculated during option (0.5 kHz + 1.0 kHz + 2.0 kHz + 3.0 kHz)/4).

Figure 1 shows also changes in the hearing thresholds for individual frequencies. It can be seen that the most serious hearing loss occurred after surgery for the highest frequencies. In turn, the most limited hearing threshold deterioration was observed for low frequencies.

In preop SA—60 dB SPL, speech discrimination ranged from 0 to 100% (median 90%). In the seven cases for which speech discrimination at 60 dB SPL was 0%, at higher levels of 70–120 dB SPL, this value reached levels of 65–100%. Analogous postop values decreased, and although they ranged from 0 to 100%, the median was 30%. In 28 cases, postop speech discrimination at a level of 60 dB SPL was 0%; however, at levels of 100–120 dB SPL, 0% was observed in 14 cases. Postoperative analysis of the deterioration of speech discrimination showed statistical significance according to the Wilcoxon test (Z = 6.60; *p* < 0.001). Detailed analysis values are included in Table 1.

Additional analysis of the distribution of speech discrimination values showed that in 26 (34.66%) patients, the postop speech discrimination values at an intensity of 60 dB SPL deteriorated by no more than 10%, while in 41 (54.66%) patients, speech discrimination did not deteriorate by more than the average deterioration of 32%. 

The analysis of the change in the distribution of hearing classes according to AAO—HNS [36] considering PTA-4 and speech discrimination for an intensity of 60 dB SPL showed that before the surgery, 47 patients were in hearing class A, and after the surgery, 24 patients were in this class. In hearing class B, the number of patients before the procedure was nine, and after the procedure, eight. In hearing class C, there were only two patients before the surgery, and only one after. In turn, in class D, after the surgery, the number of patients increased from 17 to 42, including 13 patients who suffered from complete hearing loss. A detailed quantitative distribution of hearing classes according to AAO—HNS is provided in Table 2. The AAO—HNS hearing classes before and after surgery were also analyzed using the maximum likelihood χ^2^ test, which found a statistically significant deterioration in hearing and speech discrimination at an intensity of 60 dB SPL (χ^2^= 27.54; df = 9; *p* < 0.001).

### 3.3. Characteristics of Electrophysiological Tests

#### 3.3.1. Auditory Brainstem Responses—ABR

The location of VS and the possible cochlear nerve and the brainstem compression may sometimes cause desynchronization of the ABR response, which is manifested by the disappearance of selected waves I, III, V. This is one of the reasons why individual ABR waves could not be recorded in some of the described patients. In all 75 patients, latencies of waves I and V were determined; in some cases, most often during VS resection, it was not possible to identify wave III; therefore, the I–V interlatencies, which were determined in 75 patients in each of the cases, could be used for a more accurate assessment at each of the three stages of the surgery. Nevertheless, as described in the methodology, the values of all I–III, III–V and I–V interlatencies were analyzed, and these values are presented in Table 3. The intraoperative dynamic changes of the measured ABR parameters mentioned above observed before (Pre), during (Intra) and after (Post) VS resection are expressed as the difference between Post and Pre, Post and Intra, and Intra and Pre. Before the start of the VS resection, the I–V interlatencies ranged from 4.06 to 6.15 ms, and only in three cases, this value exceeded 6 ms (average 5.06; median 4.95; min–max: 4.06–6.15; Q1 4.65; Q3 5.53; SD 0.55). During removal, the value of the I–V interlatencies ranged from 3.72 to 7.38 ms, but values above 6 ms were observed only for four cases (average 5.07; median 5.00; min–max 3.72–7.38; Q1 4.7; Q3 5.37; SD 0.61). The observed values after VS resection showed that in 2 cases, the value of the I–V interlatencies was higher than 6 ms, but in as many as 14 cases, this value was undetectable (for the purposes of this study, a value of 12.8 ms was assumed). Therefore, on average, significant extension of the I–V interlatencies’ values was observed, which reached the value of 6.43 ms (median 5.02; min–max 3.65–12.80; Q1 4.77; Q3 5.75; SD 3.11). Table 3 contains all individual values for waves I, III, V and for their interlatencies I–III, III–V, and I–V. On this basis, after tumor removal, the largest wave V was prolonged by an average of 1.29 ms and reached a value of 8.27 ms (median 7.28; min–max 6.20–12.80; Q1 = 6.85; Q3 8.05; SD 2.24).

Statistical analysis using the Wilcoxon test showed statistically significant changes in the values of waves I, III, and V before and during tumor removal as well as before and after removal of the VS, as described in detail in Table 3. In turn, changes after VS resection in relation to the surgical manipulation stage were observed only for wave V (Post vs. Intra: Z = 2.30; *p* < 0.05). Statistically significant changes were also obtained at the same time of surgery for the I–V interval (Post vs. Intra: Z = 2.08; *p* < 0.05). The Wilcoxon test also showed statistically significant changes in the values of the I–V interlatencies after surgery compared to those from before VS resection (Post vs. Pre: Z = 2.37; *p* < 0.05).

#### 3.3.2. Transtympanic Electrocochleography

TT-ECochG responses were recorded in all 75 patients at three stages of the surgery. The following intraoperative dynamic changes of the TT-ECochG parameters were measured: AP—Amp and AP—Lat observed before (Pre), during (Intra) and after (Post) VS resection are expressed as the difference between Post and Pre, Post and Intra, and Intra and Pre. AP—Lat values at the beginning of the surgery ranged from 1.55 ms to 2.63 ms (average 1.92; median 1.88; Q1 1.75; Q3 2.02; SD 0.23), while the corresponding amplitude values were 0.71 µV to 43.66 µV, respectively (average 7.93; median 4.79; Q1 43.66; Q3 2.04; SD 9.16). During intraoperative manipulations at the stage of the VS resection, AP—Lat values increased, while the AP—Amp was significantly reduced, reaching the lowest values of median 3.38 µV during the entire surgery (average 4.94; min–max: 0.14–28.71; Q1 1.56; Q3 7.10; SD 4.88). After VS resection, the greatest delay of the A—Lat occurred (average 2.89 ms; median 2.27; min–max 1.68–5.60; Q1 1.95; Q3 2.85; SD 1.38). In this stage of the surgery, a decrease in AP—Amp was also observed (average 5.00 µV; median 2.78 µV; min–max 0.00–38.94; Q1 1.23; Q3 5.57; SD 6.57). Detailed latency and amplitude values of TT-ECochG are provided in Table 3.

Statistical analysis using the Wilcoxon test showed a statistically significant increase in AP—Lat post VS resection compared to pre VS resection (Post vs. Pre: Z = 7.31; *p* < 0.001) and a reduction in AP—Amp (Z = 3.29; *p* < 0.001). In a statistical analysis using the same test, comparing Intra and Pre VS resection AP parameters, a statistically significant delay in AP—Lat (Intra vs. Pre: Z = 7.14; *p* < 0.001) and a decrease in amplitude (Z = 3.25; *p* < 0.001) were demonstrated for data recorded during VS resection. The Wilcoxon test also showed a statistically significant increase in AP—Lat values after VS resection compared to the data recorded during VS resection (Post vs. Intra: Z = 2.18; *p* < 0.005). Details are presented in Table 3.

### 3.4. Assessment of the Relationship between Audiological Tests and Electrophysiological Tests Used for Intraoperative Hearing Monitoring

**Figure 2:**
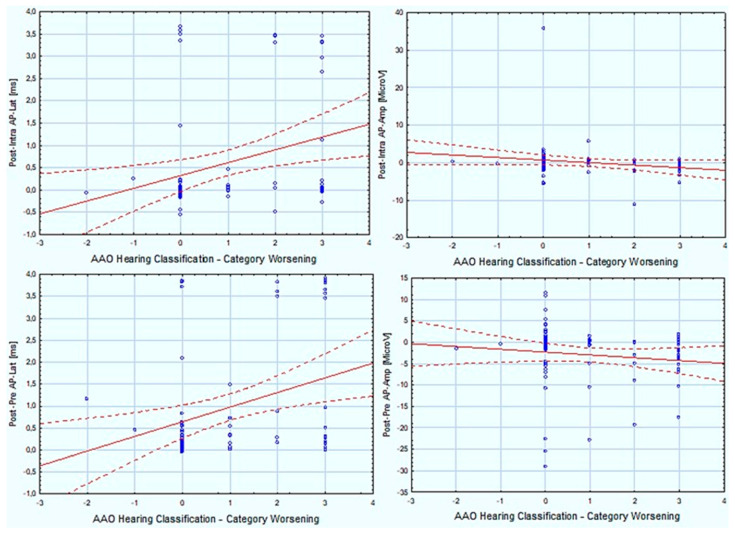
Correlation between American Academy of Otolaryngology–Head and Neck Surgery Hearing Classification (difference between post- and preoperative hearing category) and intraoperative changes of parameters describing transtympanic electrocochleography parameters (action potential latency and amplitude) during three stages of the surgery. AAO—HNS: American Academy of Otolaryngology–Head and Neck Surgery; ms: milliseconds; AP_Lat: action potential latency; AP_Amp: action potential amplitude; Post–intra: difference between results after and during surgery; Post–pre: difference between results after and before surgery.

Based on statistical analysis using the Spearman’s Test, several correlations were demonstrated between hearing deterioration after surgery expressed as Post–Pre PTA-4, deterioration of speech discrimination for SA—60 dB SPL presented as Post–Pre SA—60 dB SPL, AAO—HNS hearing classes changes also expressed as the difference between Post and Pre AAO—HNS classification (Table 4), and electrophysiological tests measured intraoperatively (ABR and TT-ECochG). Some are described below, while details are presented in Figure 2 and Table 1, Table 2, and Table 4. Spearman’s test showed a statistically significant correlation between Post and Pre PTA-4 and differences between postoperative and intraoperative values of the ABR I–V interlatencies (R = +0.39) (Post–intra ABR I–V). Additionally, a statistically significant correlation was revealed between the Post and Pre PTA-4 and differences between Pre and Post values of the ABR I–V interlatencies (R = +0.42) (Post–Pre ABR I–V). Similar results were revealed for 2 and 4 kHz in tonal audiometry (see Table 5). Analogous analyses of speech discrimination expressed as Post-Pre SA—60 dB SPL showed no significant correlations (details in Table 5).

Spearman’s Test also showed a statistically significant correlation between postoperative SA—60 dB SPL and the Post–intra ABR I–V (R = −0.23). The same tendency was observed for Post–pre ABR I–V (R = −0.19), although with no significance. Correlations were also found between postop SA—60 dB SPL and the Post–intra AP—Lat (R = −0.42) as well as for Post–intra AP—Amp (R = 0.33) (details are presented in Table 5).

The same statistical correlations found between intraoperative electrophysiological tests and hearing status were evaluated according to AAO—HNS hearing classes. The majority of tests showed significant correlation between hearing worsening expressed as a difference in AAO—HNS hearing classes before and after surgery and the prolongation of Post–intra AP—Lat (R = +0.26), which was analogous to the reduction of the postoperative AP—Amp (R = −0.3). A correlation using the same test also demonstrated dependences of AAO—HNS hearing classes before and after VS resection and prolongation of AP—Lat or reduction of AP—Amp (R = 0.19 and R = −0.24, respectively) (see details in Table 4 and Figure 2).

### 3.5. Assessment of the Relationship between ABR and TT-ECochG during Intraoperative Hearing Monitoring

**Figure 3:**
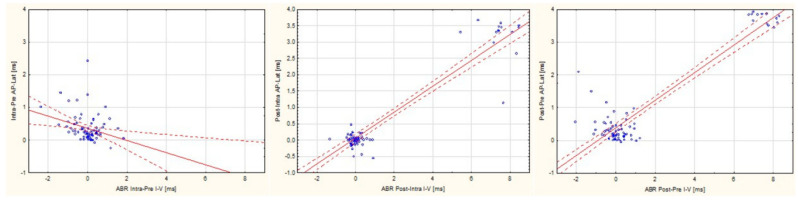
Correlation between auditory brainstem response interlatencies I–V and action potential latency changes during vestibular schwannoma resection calculated in various forms: Intra—Pre; Post—Intra and Pos—Pre. ABR I–V: auditory brainstem response interlatencies I–V; AP_Latency: action potential latency; ms: milliseconds; Intra–pre: difference between results during and before surgery; Post–intra: difference between results after and during surgery; Post–pre: difference between results after and before surgery.

Statistical analyses showed some significant correlations between ABR and TT-ECochG during IM of hearing during VS resection. Spearman’s Test revealed that the prolongation of postop AP—Lat in relation to intraoperative AP—Lat (Post–intra AP—Lat) correlates with the Post–intra ABR I–V (R = +0.4). A similar correlation analysis between Post–pre AP—Lat and Post–pre ABR I–V also revealed significance (R = +0.3). A different tendency was observed upon analysis of the correlation between Intra–pre AP—Lat and Intra–pre ABR I–V (R = −0.27). In the next analysis, Spearman’s test showed correlations between Post–intra AP-Amp and Post–intra ABR I–V (R = −0.37). Some correlations were also observed between the reduction in the amplitude of the action potential after surgery (Post–pre AP—Amp) and Post–pre ABR I–V (R = −0.23). Details are presented in Table 5 and Figure 3. 

### 3.6. Assessment of the Relationship between Audiological and Electrophysiological Tests Used for Intraoperative Hearing Monitoring and Vestibular Schwannoma Dimensions

All parameters characterizing preoperative and postoperative TA and SA as well as intraoperative measurements of ABR and TT-ECochG were correlated with VS dimensions and Koos Grading Scale classifications. Among all statistical analyses, significant correlation was revealed only in one test. Spearman’s test showed that the intraoperative prolongation of ABR I–V interlatency expressed as an Intra–pre ABR I–V correlated with the size of the vertical axis of VS (R = +0.24; t(n-2) = 2.11; *p* < 0.05). No other significant correlations were revealed.

## 4. Discussion

The most common pathological change in the cerebellopontine angle that affects hearing is VS. Its occurrence is estimated at 85–90% of tumors located in this area. Meningiomas and other cranial nerve schwannomas (of the VII or V nerve), or so-called non-acoustic CPATs, are much less frequently observed [39,40]. According to Żurek et al. the incidence of vestibular schwannoma in Poland is 1.99 cases per 100,000 people per year; the average incidence is 19.87 per 1,000,000 people and ranges from 6.41 to 35.07 depending on the age group [40]. Long-term research conducted by Larjavaar et al. in the Scandinavian countries (Denmark, Finland, Norway, and Sweden) shows that in the years 1987–2007, incidence rates ranged from 6.10 to 11.60 per 10,000,000 person years. These studies showed an increase in the incidence of VS mainly in the mid-1990s. The greatest increase in the detection of acoustic neuromas was observed in Denmark, while in Finland, it was infinitesimal [41]. In turn, other studies conducted in Denmark in 2003–2012 showed that the VS incidence rate was 22.1 per 1,000,000 person years [42].

Many authors list unilateral, progressive hearing loss, tinnitus, and transient dizziness as the main symptoms of vestibular schwannoma [40,43,44]. In the present study, only 1 out of 75 patients did not report any symptoms before the diagnosis of VS, and it was instead detected accidentally during MRI after a head injury. In turn, the remaining 74 patients most often reported the following symptoms, which occurred individually or together: unilateral sensorineural hearing loss (60%), tinnitus (66%), and transient dizziness (44%). In research conducted by Żurek et al., it has been shown that women are more predisposed to developing VS (61.46%) [40]. Additionally, in the analyzed study, women constituted a larger proportion of patients (57.33%) than men (42.67%). 

Intraoperative hearing monitoring is used to increase the likelihood of hearing preservation during resection of VS through the middle cranial fossa or retrosigmoid approach. Most often, it is performed using ABR or TT-ECochG [7,8,11,12,13,14,16,17,18,21,28]. The available literature also includes reports on IM using a combination of ABR and CNAP tests [7,9,10,12,15,16,17,19,20,22,23,24,25,26] or TT-ECochG and CNAP [7,10,12,16,17,27,29,30,31].

The ABR test assesses neuronal conduction in the peripheral part of the auditory pathway and in the brainstem. The test is widely known and non-invasive, which is why it is the most frequently used method for IM of hearing during VS resection. However, a significant limitation of the ABR method for IM is the need to average many samples (several hundred repetitions) of the recorded electrophysiological signal to obtain an optimal signal-to-noise ratio (SNR) and thus obtain clear and repeatable responses. ABR testing involves far-field recordings with low-amplitude responses, which is often challenging in the operating room due to the presence of numerous devices generating electromagnetic fields; consequently, it requires an increase in the number of averages to obtain reliable results. Thus, the ABR technique has limited value in providing information about the hearing condition in real time [12,22,25]. Although the ABR technique does not monitor auditory functions in real time (and usually, at critical moments of the tumor resection, it takes about 1 min (minimum 20–30 s) to confirm the hearing status), ABRs reflect responses from the entire auditory pathway. This enables the identification of the “disconnected ear” effect, which is observed in the case of selective damage to the cochlear part of the VIII nerve with preserved function of the spiral ganglion [11]. Despite the above-mentioned limitations of ABR tests used for IM, they have been the most frequently used method of intraoperative hearing monitoring in recent years [7,8,9,10,11,12,13,14,15,16,17,18,19,20,21,22,23,24,25,26].

Based on research conducted by Yamakami et al., it appears that TT-ECochG more often provides better IM of hearing than ABR. According to their research, during VS resection, as many as 20 out of 22 patients, representing 91% of the total, had correct responses upon IM of hearing with the use of TT-ECochG, while only 9 out of 22 patients (41%) achieved ABR responses [15]. The latency value of wave V from the moment of surgical manipulations in their study was, on average, 6.94 ms [15] and in the described work was 7.35 ms. In the present study, it was also not possible to obtain the latency values of ABR waves I, III and V in all 75 patients. Only waves I and V were identified in all subjects at each of the three stages of surgery (before tumor removal, during, and after), while wave III was legible and repeatable before VS removal in 78% of the total cases, during tumor removal in 54.67%, and at the end of the procedure in 53.3% of the operated patients. Schlakel et al. also believe that slightly more reliable IM results are obtained using TT-ECochG [8]. These authors showed similar trends in results to ours, showing that the value of the I–V interlatencies at the beginning of the procedure was on average 5.41 ms, and after tumor removal, it ranged from 5.96 to 7.85 ms. In turn, in the analyzed group, the average value of the I–V interlatencies before VS removal was 5.05 ms, and at the end of the operation, it extended to 6.42 ms. 

A test that helps to solve the problems and limitations of the ABR method during intraoperative hearing monitoring is the recording of auditory potentials from the promontory, i.e., TT-ECochG. This technique, due to the measurements of potentials close to their generation source (the near-field technique), provides electrophysiological responses that have an amplitude several times higher than ABR; therefore, it is much easier to achieve a favorable SNR coefficient to obtain a readable functional potential from the VIII nerve. Since a relatively small number of samples are required to obtain a clear, repeatable, and averaged reading, and responses are sent on average every few seconds (5–6 s), it can be assumed that monitoring using TT-ECochG takes place in real time with minimal time delay [12,27,28]. The intraoperative TT-ECochG test is characterized by high-frequency specificity, high sensitivity, and readable responses with a relatively small number of averaged repetitions (64–256). However, since the answers come only from the peripheral parts of the VIII cranial nerve, TT-ECochG is not able to register the so-called “disconnected ear” effect. Schlake et al. reported that occasionally, TT-ECochG responses were recorded intraoperatively, and postoperative hearing tests revealed that a complete hearing loss occurred in the operated ear [8]. Despite this very rarely observed phenomenon, most authors believe that real-time response registration, high sensitivity to even small changes in auditory functions, and the possibility of online analysis make TT-ECochG a very useful tool for IM during otoneurological operations [7,8,10,11,12,16,17,18,27,28]. That said, it should be remembered that TT-ECochG provides information only about the auditory nerve and cochlea; therefore, its use as the only test is not an optimal IM method. For this reason, it seems more rational to combine at least two electrophysiological test methods to conduct IM [7,10,11,12,15,16,17,27].

In most studies that analyzed the individual parameters of the action potential, i.e., amplitude and latency, during intraoperative hearing monitoring, frequently repeated results were observed, namely that after VS resection, the AP amplitude was reduced, and its latency was prolonged [7,8,10,11,12,16,17,18,27,28]. Yamakami et al. [15] and Colletti et al. [7] described changes in the morphology of TT-ECochG, i.e., the AP amplitude decreased slightly (by 1 μV), and the latency increased by 0.07 ms. Similar changes were observed by Morawski et al. [28], i.e., the amplitude did not decrease by more than 25%, and the latency did not extend beyond 0.2 ms. In a 2016 study analyzing 15 patients, Pobożny et al. [11] noted that the AP latency value before tumor removal was 2.15 ms, and at the end of the operation, it increased to 2.73 ms. In turn, the amplitude of the action potential decreased from an initial value of 1.94 µV to 1.43 µV. In the currently studied group, this time analyzing 75 patients, an extension of the AP latency from 1.92 ms to 2.89 ms after the operation was also demonstrated; we also found a simultaneous decrease in its amplitude from before the start of VS removal (7.93 µV) to the value obtained at the end of the operation (4.94 µV).

In most of the scientific studies conducted, a characteristic common feature of the results is that hearing impairment or complete deafness are common phenomena in patients after the VS removal surgery, even if it was performed with the use of intraoperative hearing monitoring [7,8,9,11,15,24,25,28]. During this type of surgery, the morphology of the ABR or TT-ECochG response may change at different stages of the surgery, which often translates into poor postoperative audiological test results. Schlake et al. in their study showed that there was no correlation between postoperative audiological results and the latency values of ABR waves I, III, V. In turn, a highly significant correlation was detected between pre- and postoperative AP latency values of hearing before and after VS removal [8]. A study by Morawski et al. [28] showed a high correlation between intraoperative changes in the morphology of TT-ECochG and the postoperative hearing threshold (R = +0.93; *p* < 0.0001). This study, using Spearman’s test, showed a correlation between changes in the distribution of AAO—HNS hearing classes before and after the surgery and a decrease in the amplitude of the action potential (R = −0.24; *p* < 0.05) and an increase in AP latency relative to the moment of its removal (R = 0.26; *p* < 0.05). Changes in the morphology of TT-ECochG and ABR caused by surgical manipulations during tumor removal are a common phenomenon [7,8,9,11,24,25,27]. Particularly precarious moments of the operation are bleeding from the tumor and the need to use bipolar coagulation [15,24,28]. It has also been observed that after long-term milling of the internal auditory canal or traction of the auditory nerve during tumor removal, changes in the morphology of the TT-ECochG response occur [7,15,24,25] with a subsequent decrease in the AP amplitude and/or extension of latency [7,11,15,28]. The above events translate to substantially poorer audiological results after surgery [10,11,27]. Based on research conducted in 2016 by Pobożny et al., a correlation was detected: with the postoperative extension of AP latency, the hearing threshold deteriorates, and the action potential amplitude decreases [11]. Similar results were also obtained in the presented work, as discussed in the Results section.

There are many studies dedicated to the preservation of hearing in patients undergoing surgery for VS using intraoperative hearing monitoring. To analyze changes in audiological results and predict hearing preservation after surgery, authors most often use hearing classes according to AAO—HNS [6,9,15,17,19,20,22,45,46] or the Garden–Robertson Scale [47], or they assess hearing preservation using hearing threshold values (PTA) equal to or better than 40 dB and a speech detection threshold (SDT) of 70% or better [38]. Many factors influence the preservation of hearing in patients after VS removal surgery. Morawski et al. [10] in their work included the choice of surgical approach, preoperative hearing threshold no worse than 50 dB HL at 1000 Hz, PTA-4 better than 60 dB HL, and speech intelligibility no worse than 60% at an intensity of 60 dB SPL. They mention, among the factors determining postoperative hearing preservation, the size of the tumor and its location in relation to the cerebellopontine angle (smaller tumors that are intraductal and less than 20 mm in the long axis allow for a greater chance of preserving hearing). Similar observations were also described by Concheri et al. [48]. The use of intraoperative hearing monitoring during surgery also has a tremendous impact on increasing the probability of hearing preservation in patients operated on for VS. Factors influencing postoperative hearing preservation also include age below 50 years, female gender, and the place of tumor origin (the lower or upper vestibular nerve) [2,10,48].

In their study, Yancey et al. analyzed 130 patients, including 45 patients operated on using the MFA. In 55.6% of patients, hearing was maintained at a useful hearing level (hearing class A/B according to AAO—HNS) [45]. In the analyzed study, after surgery, 32 patients (42.67%) were in hearing classes A and B, and before surgery, 56 patients were in these classes (74.67%). In turn, another study [46] showed that 49 out of 50 patients included in the study were classified as hearing classes A and B, and after surgery, 37 of them remained in the same hearing classes. In the presented study, the average hearing threshold for 75 patients before surgery was 25.02 ± 15.53 dB HL, and after surgery, it decreased by 30.03 dB HL. Interestingly, in a similar study by the same authors, but in a smaller study group (15 patients), it was also shown that the hearing threshold deteriorated postoperatively by 30.07 dB HL [11]. Kosty et al. analyzed a group of 63 patients operated on for VS through the middle cranial fossa approach. Based on preoperative analysis according to AAO—HNS, 32 (52%) patients were in hearing class A, 15 (24%) were in class B, 9 (14%) were in class C, and only 5 (9%) were in hearing class D. In the postoperative analyses, 5 patients from class D were excluded, so that only those whose hearing was at least at a useful level remained. Of the group of patients from classes A-C, in one from class B, postoperative results showed improvement, and the patient was transferred to hearing class A. A total of 18 patients from hearing classes A-C remained in their preoperative classes. In six of them, their postoperative results worsened by one class. However, 24 patients from preoperative hearing classes A-C qualified for hearing class D after surgery. In their opinion, the MFA provides good control during the removal of the VS, facial nerve, and VIII nerve, which translates into good postoperative hearing preservation results. They believe that even in the case of patients with less useful hearing, they can derive auditory benefits from preserved low frequencies [23]. In the analyzed group of 75 patients operated on due to VS, 47 of them were in hearing class A before the operation, and after VS removal, 23 of them remained in this class. Additionally, in one patient from preoperative hearing class B, the audiological results improved after the procedure and allowed the patient to qualify for hearing class A. Seven patients from preoperative hearing classes A-B obtained worse hearing tests results which placed them down one class (A->B—6 people; B->C—1 person). Interestingly, in one patient who qualified for hearing class D before the surgery, the results improved by two classes after the surgery, and the patient was transferred to class B. As we know, the operation to remove VS carries the risk of hearing deterioration or complete hearing loss; even with the use of intraoperative hearing monitoring in 18 patients from hearing class A, after the surgery, significantly poorer audiological results were obtained, and they were therefore moved into hearing class D. From hearing class B, six patients moved to class D, and from the preoperative hearing class C, two people moved to class D. Sixteen patients remained in hearing class D after surgery. In the described group, 13 patients completely lost their hearing functions after surgery. Six of them were classified in group A before the operation, three of them were in hearing class B, and four were in class D. Despite different postoperative results, 62 patients (82.67%) had preserved hearing after surgery (hearing classes A-D), and 33 (44%) of them still had useful hearing.

Hearing deterioration or complete hearing loss is one of the most common complications of VS removal surgery [7,8,9,11,12,15,20,23,24,25,28]. Thanks to the continuous development of medicine and bioengineering, patients can receive help in the form of various types of hearing aids. The hearing rehabilitation process is extremely important for all VS patients’ hearing, the improvement of which translates into a better quality of life. Currently, patients after VS removal surgery who have hearing loss or deterioration as a result of the surgery can be provided with numerous methods supporting auditory rehabilitation. Among them, we can distinguish typical hearing aids, hearing aids with contralateral routing of signal (CROS) or bilateral contralateral routing of signal (BiCROS), bone-anchored hearing systems (BAHSs), and cochlear implants (CIs) [49]. Thanks to the growing number of new methods and hearing supporting devices, post-VS-surgery patients, in the event of hearing deterioration, do not have to worry that they have lost their hearing irreversibly. It is important to offer all patients the best possible treatment method for VS and to provide the best possible assistance after surgery in terms of auditory, facial nerve, and balance organ rehabilitation.

## 5. Conclusions

IM of hearing using ABR, TT-ECochG increases the chance of preserving hearing in patients during VS resection. It is worth remembering that despite the use of IM of hearing during VS resection, there is no full guarantee that hearing will not be affected. Choosing a combination of IM methods, such as ABR + TT-ECochG, as well as good communication between the surgeon and the audiologist intraoperatively monitoring hearing will significantly improve audiological postoperative results because quickly detecting abnormalities in the cochlea, the eighth nerve, and the auditory pathway will allow us to avoid irreversible changes. In the last few years, current guidelines have emerged, confirming that monitoring of the function of the VIII nerve should be used during VS resection when an attempt is made to preserve hearing [6,50].

## Figures and Tables

**Table 1:** Results of descriptive statistical analyses for tonal audiometry calculated using the following formula: (0.5 kHz + 1.0 kHz + 2.0 kHz + 3.0 kHz)/4)(PTA-4) and speech audiometry presented at an intensity of 60 dB SPL obtained before and after surgery in 75 patients included in the study.

	Ave	Med	Range	Q1–Q3	SD	Test Wilcoxona
Pre PTA-4 [dB HL]	25.02	22.50	2.50–63.75	11.25–37.50	15.53	
Post PTA-4 [dB HL]	55.05	43.75	10.00–130.00	22.50–70.00	39.48	
Post–Pre PTA-4 [dB]	30.03	15.00	−6.25–126.25	6.25–38.75	36.46	Z = 7.20 *; *p* < 0.001
Pre Speech Audiometry 60 dB SPL [%]	70.93	90.00	00.00–100.00	50.00–100.00	33.68	
Post Speech Audiometry 60 dB SPL [%]	38.93	30.00	0.00–100.00	0.00–80.00	37.42	
Post–Pre Speech Audiometry 60 dB SPL [%]	−32.00	−20.00	−100.00–45.00	−55.00–5.00	32.40	Z = 6.60 *; *p* < 0.001

PTA-4: tonal audiometry calculated using the following formula (0.5 kHz + 1.0 kHz + 2.0 kHz + 3.0 kHz)/4); dB HL: decibel hearing level; dB SPL: decibel sound pressure level; Pre: before surgery; Post: after surgery; Post–Pre: difference between results after and before surgery (for PTA-4, a positive value—hearing deterioration; negative—hearing improvement, and for speech audiometry with 60 dB SPL, a negative value means deterioration of speech discrimination, and positive means improvement in speech discrimination). (*): statistically significant; Ave: average (mean); Med: median; Range: min–max; Q1–Q3: first quartile–third quartile; SD: standard deviation.

**Table 2:** Hearing classes according to the American Academy of Otolaryngology—Head and Neck Surgery Hearing Classification for the tonal audiometry calculated in option (0.5 kHz + 1.0 kHz + 2.0 kHz + 3.0 kHz)/4)(PTA-4) and speech discrimination (60 dB SPL) obtained via pre- and postoperative hearing tests in 75 patients operated upon for vestibular schwannoma through the middle fossa approach.

AAO—HNS Hearing Classification	Pre PTA-4	Post PTA-4	Pre Speech Audiometry 60 dB SPL	Post Speech Audiometry 60 dB SPL	Pre PTA-4 + Speech Audiometry 60 dB SPL	Post PTA-4 + Speech Audiometry 60 dB SPL
Class A	50 (66.67%)	24 (32.00%)	32 (42.67%)	24 (32.00%)	47 (62.67%)	24 (32.00%)
Class B	20 (26.67%)	20 (26.67%)	6 (8.00%)	8 (10.67%)	9 (12.00%)	8 (10.67%)
Class C	5 (6.67%)	15 (20.00%)	12 (16.00%)	1 (1.33%)	2 (2.66%)	1 (1.33%)
Class D	0 (0.00%)	16 (21.33%)	25 (33.33%)	42 (56.00%)	17 (22.67%)	42 (56.00%)
χ^2^ Test Pre vs. Post	χ^2^ = 27.54; df = 9; *p* < 0.001					

AAO—HNS: American Academy of Otolaryngology–Head and Neck Surgery; PTA-4: tonal audiometry calculated in option (0.5 kHz + 1.0 kHz + 2.0 kHz+ 3.0 kHz)/4); Pre: before surgery; Post: after surgery.

**Table 3:** Latencies of waves I, III, V and interlatencies I–III, III–V, I–V of auditory brainstem responses and values of action potential latency and amplitude of transtympanic electrocochleography obtained at the beginning, during and at the end of the surgery for vestibular schwannoma resection through the middle fossa approach.

	N	Ave	Med	Range	Q1–Q3	SD	Test Wilcoxona
Pre ABR I/III/V [ms]	75/60/75	1.92/4.85/6.98	1.88/4.71/6.92	1.55–2.63/ 3.98–6.1/ 5.88–8.38	1.75–2.02/ 4.44–5.25/ 6.5–7.42	0.23/0.52/0.58	
Pre ABR I–III/ III–V/I–V [ms]	60/60/75	2.95/2.06/5.05	2.79/2.05/4.95	2.10–4.00/ 1.32–2.8/ 4.06–6.15	1.75–2.02/ 2.60–3.32/ 4.65–5.53	0.49/0.27/0.55	
Intra ABR I/III/V [ms]	75/42/75	2.28/5.22/7.35	2.13/5.10/7.25	1.68–4.47/ 4.17–7.83/ 6.20–9.68	2.00–2.38/ 4.67–5.65/ 6.85–7.78	0.48/0.73/0.71	Pre vs. Intra I: Z = 7.17 *; *p* < 0.001 Pre vs. Intra III: Z = 3.28 *; *p* < 0.01 Pre vs. Intra V: Z = 4.63 *; *p* < 0.001
Intra ABR I–III/ III–V/I–V [ms]	42/42/75	3.05/2.04/5.07	2.94/2.03/5.00	1.95–5.53/ 1.55–2.86/ 3.72–7.38	2.70–3.37/ 1.83–2.22/ 4.70–5.37	0.63/0.29/0.61	
Post ABR I/III/V [ms]	61/40/75	2.26/5.11/8.27	2.15/5.01/7.28	1.68–4.17/ 4.15–6.58/ 6.22–12.80	1.90–2.52/ 4.64–5.49/ 6.85–8.05	0.47/0.58/2.24	Pre vs. Post I: Z = 6.45 * *p* < 0.001 Pre vs. Post III: Z = 2.54 *; *p* < 0.05 Pre vs. Post V: Z = 5.22 *; *p* < 0.001
Post ABR I–III/ III–V/I–V [ms]	40/40/75	2.92/2.05/6.42	2.88/2.02/5.02	1.95–3.97/ 1.65–2.95/ 3.65–12.80	2.63–3.11/ 1.90–2.21/ 4.77–5.75	0.43/0.26/3.01	Pre vs. Post I–V: Z = 2.37 *; *p* < 0.05 Intra vs. Post I–V: Z = 2.08 *; *p* < 0.05
ABR I–V Intra–pre [ms]	75	0.02	0.06	−2.35–1.83	−0.31–0.37	0.66	
ABR I–V Post–intra [ms]	75	1.36	0.06	−3.04–8.48	−0.15–0.42	3.01	
ABR I–V Post–pre [ms]	75	1.37	0.13	−2.03–8.30	−0.25–0.91	3.03	
Pre TT-ECochG Latency [ms]	75	1.92	1.88	1.55–2.63	1.75–2.02	0.23	
Pre TT-ECochG Amplitude [µV]	75	7.93	4.79	0.71–43.67	2.04–10.50	9.16	
Intra TT-ECochG Latency [ms]	75	2.28	2.13	1.68–4.47	2.00–2.38	0.48	Pre vs. Intra Lat: Z = 7.13 *; *p* < 0.001
Intra TT-ECochG Amplitude [µV]	75	4.94	3.38	0.14–28.71	1.56–7.10	4.87	Pre vs. Intra Amp: Z = 3.25 *; *p* < 0.01
Post TT-ECochG Latency [ms]	75	2.89	2.27	1.68–5.60	1.95–2.85	1.38	Pre vs. Post Lat: Z = 7.39 *; *p* < 0.001 Intra vs. Post Lat: Z = 2.82 *; *p* < 0.01
Post TT-ECochG Amplitude [µV]	75	5.00	2.78	0.00–38.94	1.23–5.57	6.57	Pre vs. Post Amp: Z = 3.29 *; *p* < 0.01

ABR: auditory brainstem responses; TT-ECochG: transtympanic electrocochleography; ABR I/III/V: auditory brainstem response latencies of wave I/III/V; ABR I–III/III–V/I–V: auditory brainstem response interlatencies I–III/III–V/I–V; ms: milliseconds; µV: millivolts; Pre: before surgery; Intra: during surgery; Post: after surgery; Intra–pre: difference between results during and before surgery (for ABR/TT-ECochG, a positive value—prolongation of latency/interlatencies value/s; negative—reduction of latency/interlatencies value/s and for TT-ECochG, a positive value—increase in amplitude; negative—a decrease in amplitude); Post–pre: difference between results after and before surgery; Post–intra: difference between results after and during surgery. (*): statistically significant; N: total number of research group; Ave: average (mean); Med: median; Range: min–max; Q1–Q3: first quartile–third quartile; SD: standard deviation.

**Table 4:** Spearman test revealing correlations between intraoperative transtympanic electrocochleography parameters (action potential latency and amplitude) measured in three stages of surgery (pre-, intra- and post-vestibular schwannoma resection) and postoperative hearing worsening evaluated according to the American Academy of Otolaryngology–Head and Neck Surgery Hearing Classification in 75 patients included in the study.

AAO—HNS Classification Hearing Worsening	N	R	t(n-2)	*p*-Value
AAO—HNS Hearing Worsening vs. TT-ECochG_Intra–pre_AP-Lat	75	0.07	0.64	*p* > 0.05
AAO—HNS Hearing Worsening vs. TT-ECochG_Intra–pre_AP-Amp	75	−0.21	−1.86	0.067
AAO—HNS Hearing Worsening vs. TT-ECochG_Post–intra_AP-Lat	75	0.26 *	2.28	*p* < 0.05
AAO—HNS Hearing Worsening vs. TT-ECochG_Post–intra_AP-Amp	75	−0.30 *	−2.68	*p* < 0.01
AAO—HNS Hearing Worsening vs. TT-ECochG_Post–pre_AP-Lat	75	0.21	1.8	0.075
AAO—HNS Hearing Worsening vs. TT-ECochG_Post–pre_AP-Amp	75	−0.24 *	−2.15	*p* < 0.05

AAO—HNS: American Academy of Otolaryngology—Head and Neck Surgery; TT-ECochG: transtympanic electrocochleography; AP_Lat: action potential latency; AP_Amp: action potential amplitude; Intra–pre: difference between results during and before surgery; Post–intra: difference between results after and during surgery; Post–pre: difference between results after and before surgery; N: total number in the research group. (*): statistically significant. Statistical analyses were performed using the Spearman test.

**Table 5:** Correlations between audiological tests performed before and after surgery and intraoperatively measured auditory brainstem responses and transtympanic electrocochleography parameters action potential latency and amplitude measured in three stages of the surgery (Pre-, Intra- and Post-vestibular schwannoma resection) and hearing postoperative worsening evaluated according to American Academy of Otolaryngology–Head and Neck Surgery Hearing Classification.

ABR, TT-ECochG and Hearing Changes	N	R	t(n-2)	*p*-Value
Post–intra ABR I–V and PTA-4 diff	75	0.39	3.62	<0.001
Post–intra ABR I–V and Post–pre 2 kHz	75	0.34	3.05	<0.01
Post–intra ABR I–V and Post–pre 4 kHz	75	0.38	3.56	<0.001
Post–pre ABR I–V and PTA-4 diff	75	0.42	3.99	<0.001
Post–pre ABR I–V and Post–pre 2 kHz	75	0.41	3.82	<0.001
Post–pre ABR I–V and Post–pre 4 kHz	75	0.44	4.23	<0.001
Intra–pre ABR I–V and AAO—HNS 60 diff	75	−0.03	−0.22	>0.05
Post–intra ABR I–V and AAO—HNS 60 diff	75	0.19	1.67	>0.05
Post–pre ABR I–V and AAO—HNS 60 diff	75	0.15	1.30	>0.05
Post–intra AP_Lat and Post SA 60 dB SPL	75	−0.42	−3.92	<0.001
Post–intra AP_Amp and Post SA 60 dB SPL	75	0.33	3.03	<0.01
Post–pre AP_Lat and Post SA 60 dB SPL	75	−0.36	−3.34	<0.01
Post–pre AP_Amp and Post SA 60 dB SPL	75	0.31	2.84	<0.01
Intra–pre AP_Lat and ABR Intra–pre I–V	75	−0.27	−2.38	<0.05
Post–intra AP_Lat and ABR Post–intra I–V	75	0.4	3.73	<0.001
Post–pre AP_Lat and ABR Post–pre I–V	75	0.3	2.70	<0.01
Post–intra AP_Amp and ABR Post–intra I–V	75	−0.37	−3.45	<0.001
Post–pre AP_Amp and ABR Post–pre I–V	75	−0.23	−2.06	<0.05
Intra–pre AP_Amp and ABR Intra–pre I–V	75	0.09	0.78	>0.05

ABR: auditory brainstem responses; TT-ECochG: transtympanic electrocochleography; ABR I–V: auditory brainstem response interlatencies I–V; diff: difference; AP_Latency: action potential latency; AP_Amp: action potential amplitude; PTA-4: tonal audiometry calculated using the following formula: (0.5 kHz + 1.0 kHz + 2.0 kHz+ 3.0 kHz)/4). SA: speech audiometry; AAO—HNS: American Academy of Otolaryngology–Head and Neck Surgery; dB SPL: decibel sound pressure level; Intra–pre: difference between results during and before surgery; Post–intra: difference between results after and during surgery; Post–pre: difference between results after and before surgery; N: total number in the research group. Statistical analyses were performed using the Spearman Test.

## Data Availability

Dataset available on request from the authors.
